# Maternal acute and chronic inflammation in pregnancy is associated with common neurodevelopmental disorders: a systematic review

**DOI:** 10.1038/s41398-021-01198-w

**Published:** 2021-01-21

**Authors:** Velda X. Han, Shrujna Patel, Hannah F. Jones, Timothy C. Nielsen, Shekeeb S. Mohammad, Markus J. Hofer, Wendy Gold, Fabienne Brilot, Samantha J. Lain, Natasha Nassar, Russell C. Dale

**Affiliations:** 1 Kids Neuroscience Centre, The Children’s Hospital at Westmead, Faculty of Medicine and Health, University of Sydney, Sydney, NSW Australia; 2 Khoo Teck Puat-National University Children’s Medical Institute, National University Health System, Singapore, Singapore; 3 The Children’s Hospital at Westmead Clinical School, Faculty of Medicine and Health, University of Sydney, Sydney, NSW Australia; 4 Department of Neuroservices, Starship Children’s Hospital, Auckland, New Zealand; 5 Child Population and Translational Health Research, Children’s Hospital at Westmead Clinical School, Faculty of Medicine and Health, The University of Sydney, Sydney, NSW Australia; 6 The Brain and Mind Centre, The University of Sydney, Sydney, NSW Australia; 7 School of Life and Environmental Sciences and Charles Perkins Centre, The University of Sydney, Sydney, NSW Australia; 8 The University of Sydney, School of Medical Sciences and Discipline of Child and Adolescent Health, Faculty of Medicine and Health, Sydney, NSW Australia; 9 Molecular Neurobiology Research Laboratory, Kids Research, Children’s Hospital at Westmead, and The Children’s Medical Research Institute, Westmead, NSW Australia; 10 Kids Neuroscience Centre, Kids Research, Children’s Hospital at Westmead, Westmead, NSW Australia; 11 School of Medical Sciences, Discipline of Applied Medical Science, Faculty of Medicine and Health, The University of Sydney, Sydney, NSW Australia

**Keywords:** Autism spectrum disorders, ADHD

## Abstract

Inflammation is increasingly recognized as a cause or consequence of common problems of humanity including obesity, stress, depression, pollution and disease states such as autoimmunity, asthma, and infection. Maternal immune activation (MIA), triggered by both acute and systemic chronic inflammation, is hypothesized to be one of the mechanisms implicated in the pathogenesis of neurodevelopmental disorders (NDD). Although there is substantial preclinical evidence to support the MIA hypothesis, the human evidence is disparate. We performed a systematic review on human studies examining associations between maternal inflammatory states and offspring NDDs (autism spectrum disorder- ASD, attention deficit hyperactivity disorder-ADHD, Tourette syndrome-TS). 32 meta-analyses and 26 additional individual studies were identified. Maternal states associated with ASD include obesity, gestational diabetes mellitus, pre-eclampsia, pollution, stress, depression, autoimmune diseases, and infection. Maternal states associated with ADHD include obesity, pre-eclampsia, smoking, low socioeconomic status (SES), stress, autoimmune disease, and asthma. Maternal states associated with TS include low SES, depression, and autoimmune diseases. Diverse maternal inflammatory states in pregnancy are associated with common offspring NDDs. Given the increased prevalence of NDDs, there is urgent need to explore relative and cumulative maternal risk factors and disease mechanisms. Defining preventable risk factors in high-risk pregnancies could mitigate the expression and severity of NDDs.

## Introduction

Systemic chronic inflammation (SCI) is implicated in many common disease states encountered throughout life including cardiovascular diseases, chronic kidney disease, autoimmune diseases, cancer, depression, and neurodegenerative diseases^1^. The overarching characteristic of SCI is a persistent, sterile, non-resolving inflammation that increases with age^1^. This contrasts with acute inflammation that is usually infection provoked and results in short-term, high-grade inflammation^1^. There is increasing evidence that environmental and lifestyle factors including obesity, unhealthy diet, psychosocial stress, physical inactivity, disturbed sleep, microbial dysbiosis, and exposure to toxicants like smoke and pollution, collectively known as the exposome, contribute to SCI^1^. Parental SCI and disease risk may be transmitted to their offspring via a “DNA inflammatory signature” through epigenetic alterations, resulting in increased risk of inflammatory diseases in the next generation^1^. Furthermore, maternal SCI in pregnancy, which is a sensitive window of immune vulnerability, may adversely affect programming of the fetal immune, metabolic and neurological systems with long-lasting effects into adulthood^1,2^.

Dynamic genetic, epigenetic, and environmental interactions play a major role in supporting and shaping neural networks in the brain throughout life^3^. Preclinical data show that early life environmental disruptions to the developing central nervous system can result in negative consequences on neurobehavioral, cognitive, and mental health outcomes in the individual^4^. During embryogenesis and fetal growth, a complex network of neural circuits is being established through glia and neuron proliferation, migration, programmed cell death, formation of synapses, and myelination^3^. Ill-timed disruption of the *in utero* environment by maternal neuroendocrine, autonomic nervous system, inflammatory, neurotropic, and metabolic factors have been shown to affect fetal neurodevelopment^4^. Initial epidemiological studies established a link between infection during pregnancy and increased risk of schizophrenia and autism spectrum disorders in children, and animal models have since discovered that the neurobehavioral sequelae result from the maternal immune response to the pathogen rather than direct invasion of the virus^1,4,5^. Thus, maternal acute inflammation during pregnancy is posited to play a key role in the pathogenesis of neurobehavioral and psychiatric disorders, amongst other pathways^4^. However, the impacts of maternal SCI in pregnancy on fetal neuro-programming is less studied or established.

**Fig. 1:**
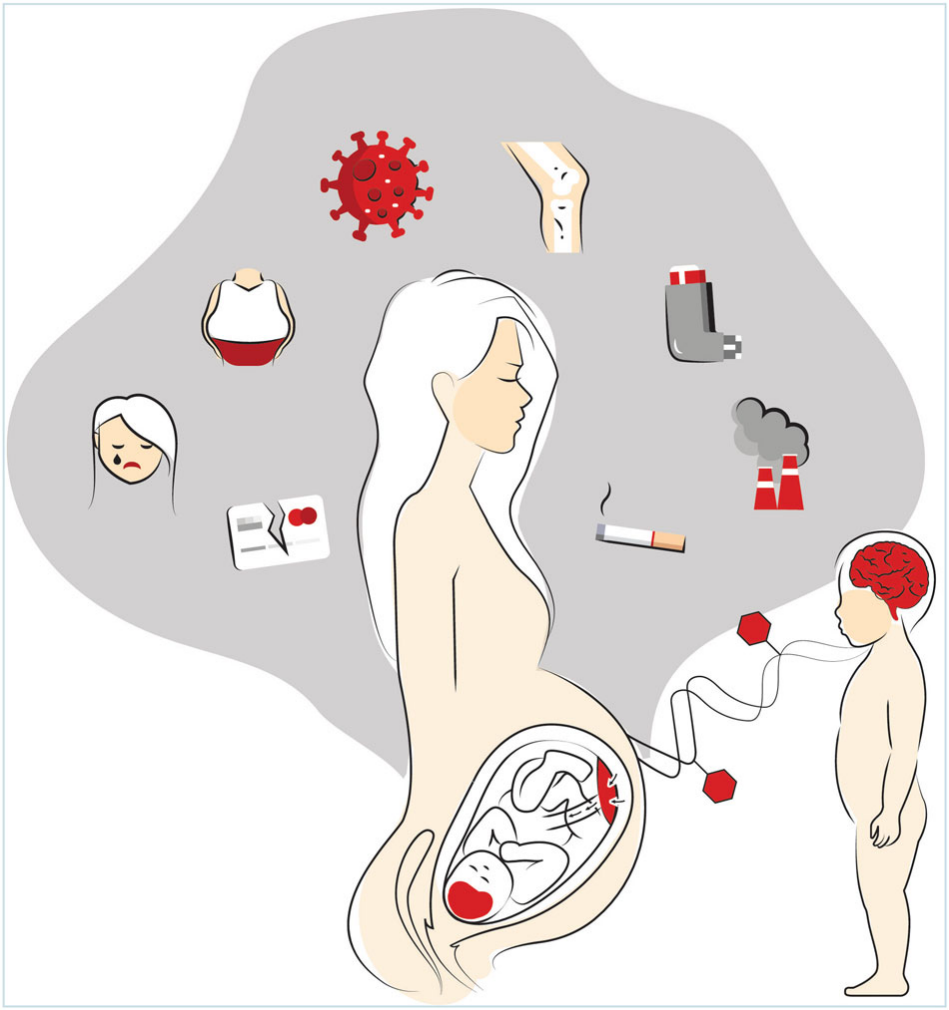
Maternal immune activation, triggered by acute and systemic chronic inflammation, is proposed to affect fetal neurodevelopment, through inflammatory and epigenetic mechanisms. Common maternal disease and environmental factors including obesity, gestational diabetes, pre-eclampsia, smoking, pollution, low socioeconomic status, depression, psychosocial stress, autoimmune diseases and asthma are implicated in systemic chronic inflammation. In addition, infection is involved in acute inflammation. These maternal inflammatory states play a key role in immune activation during pregnancy through the placenta and immature blood-brain barrier to cause dysfunction in the developing fetal brain and prime the child to be susceptible to future hits through microglia activation and epigenetic alterations, manifesting a spectrum of diverse neurodevelopmental outcomes with varied expression and progression.

Although the initial interest was centered around infection-triggered maternal immune activation (MIA), there is emerging evidence that maternal disorders associated with SCI can also trigger an abnormal immune response during pregnancy resulting in increased neurodevelopmental risks in offspring^1^. Multiple factors, which have in common SCI, including obesity, gestational diabetes (GDM), pre-eclampsia, smoking, exposure to pollution, low socioeconomic status (SES), depression, stress, autoimmune diseases, asthma and infection have been proposed to contribute to maternal immune activation (Fig. 1)^1^. Animal models have demonstrated maternal factors including obesity, stress, exposure to pollution, asthma, and infection, result in behavioral and transcriptional changes in offspring, through cytokine signaling mechanisms mediated by the placenta^4,6–8^. However, the human evidence is disparate and limited to individual inflammatory states with specific neurodevelopmental disorders.

In this systematic review, we focus on the associations between maternal inflammation in pregnancy, which are associated with acute inflammation or SCI, and common neurodevelopmental disorders in children, namely autism spectrum disorder (ASD), attention deficit hyperactivity disorder (ADHD), and Tourette syndrome (TS). Maternal infection during pregnancy was chosen as a marker for acute inflammation. In this review, we included maternal factors known to contribute to SCI such as obesity, psychosocial stress, low socioeconomic status, exposure to smoking and pollution, and human disorders known to be associated with SCI such as autoimmune disorders, asthma, depression, pre-eclampsia, and gestational diabetes. We did not include other factors in the exposome including diet, sleep, exercise, microbiome alterations, or exposure to other toxicants, as they are difficult to quantify objectively. In this review, we group these 11 risk factors as “maternal inflammatory states”. Although there have been multiple epidemiological studies linking individual maternal inflammatory states to neurodevelopmental disorders, a collective review has not been performed before. We provide evidence in human studies to support the hypothesis that a broad spectrum of maternal inflammatory states is associated with an increased risk of diverse neurodevelopmental disorders in offspring. We also discuss potential underlying mechanisms and offer recommendations for future studies.

## Materials and Methods

**Fig. 2:**
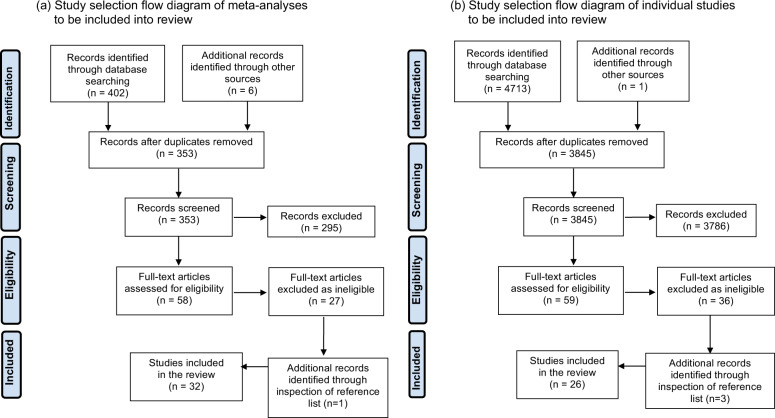
Study selection flow diagram of studies included in review. Study selection flow diagram of (**a**) meta analysis and (**b**) individual studies. No meta-analyses investigated associations between maternal asthma and ASD or ADHD, low socioeconomic status and ASD, or maternal infection and ADHD in offspring. A second search to look for individual states examining these associations was performed and included into the review.

In this systematic review, a literature search on PubMed and Embase databases was performed to identify studies. We describe the search strategy (Supplementary Table 1), study eligibility (Supplementary Table 1), search terms (Supplementary Table 2), and definitions of maternal inflammatory states (Supplementary Table 3) separately. 408 meta-analyses were identified using the first search strategy and 32 meta-analyses were included in the review after removing duplicates, irrelevant studies, and assessing full-text articles (Fig. 2). Meta-analyses of an association between individual maternal inflammatory states and ASD (21 meta-analyses comprising 146 individual study results) or ADHD (14 meta-analyses comprising 126 individual study results) in offspring were included, however, no meta-analyses were found relating to maternal inflammatory states and TS. No meta-analyses investigated associations between maternal asthma and ASD or ADHD, low SES and ASD, or maternal infection and ADHD in offspring.

**Fig. 3:**
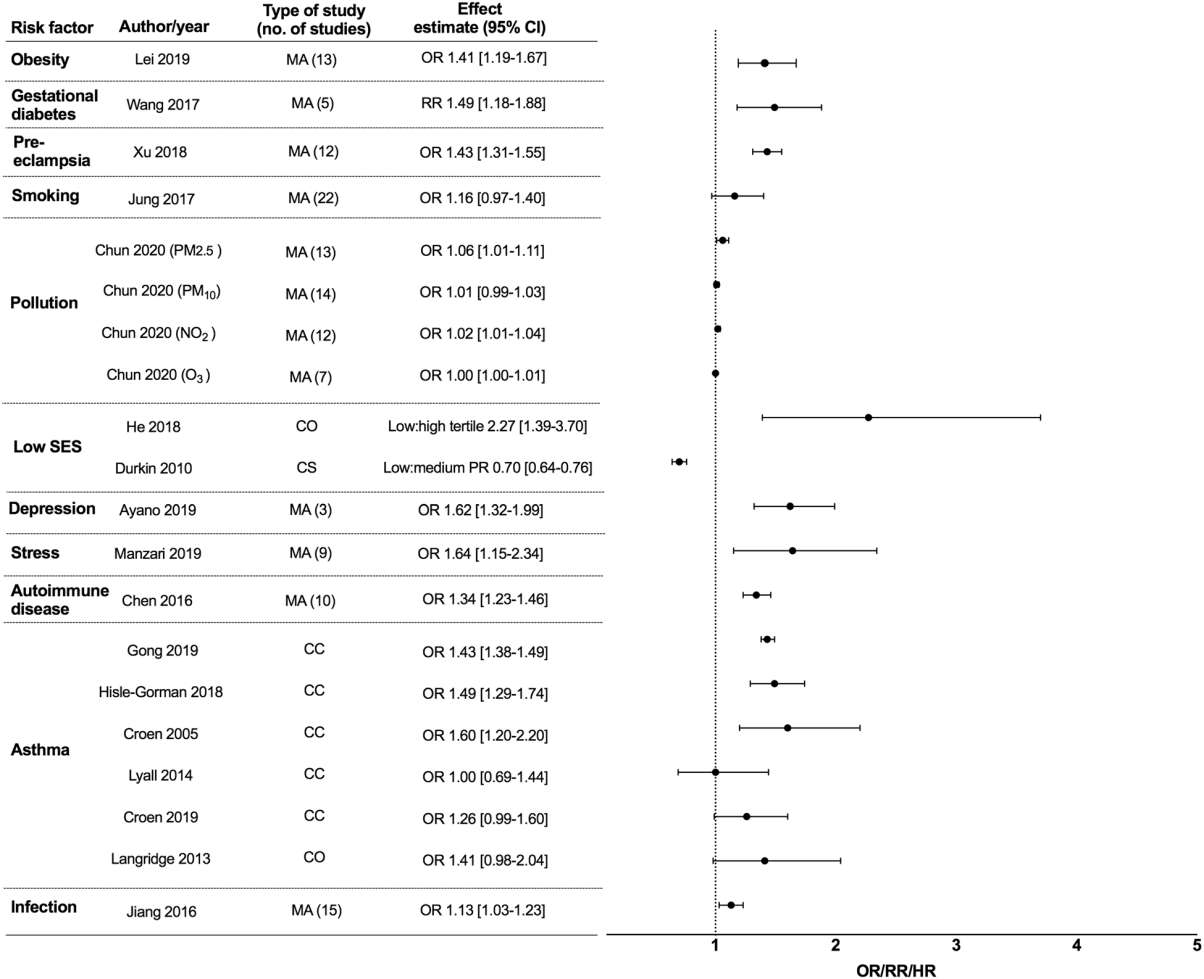
Epidemiological studies of maternal inflammatory states and autism spectrum disorder (ASD) in offspring. Maternal inflammatory states included in Y-axis (with columns including author names, year of publication, type of study, number of individual studies included in meta-analysis, effect estimate with 95% confidence interval (CI)). Effect estimate with 95% confidence interval reported in X-axis. In this forest plot, we only included the largest meta-analysis that examined the association of individual maternal inflammatory state and ASD in offspring. For full data see supplementary Fig. 1. SES = socioeconomic status, PM_2.5_ = particulate matter (particles with diameter of 2.5 micrometers or less), PM_10_ = particulate matter (particles with diameter of 10 micrometers or less), NO_2_ = nitrogen dioxide, O_3_ = ozone, MA = meta-analysis, CC = case-control, CO = cohort, CS = cross sectional, RR = relative risk, OR = odds ratio PR = prevalence ratio.

**Fig. 4:**
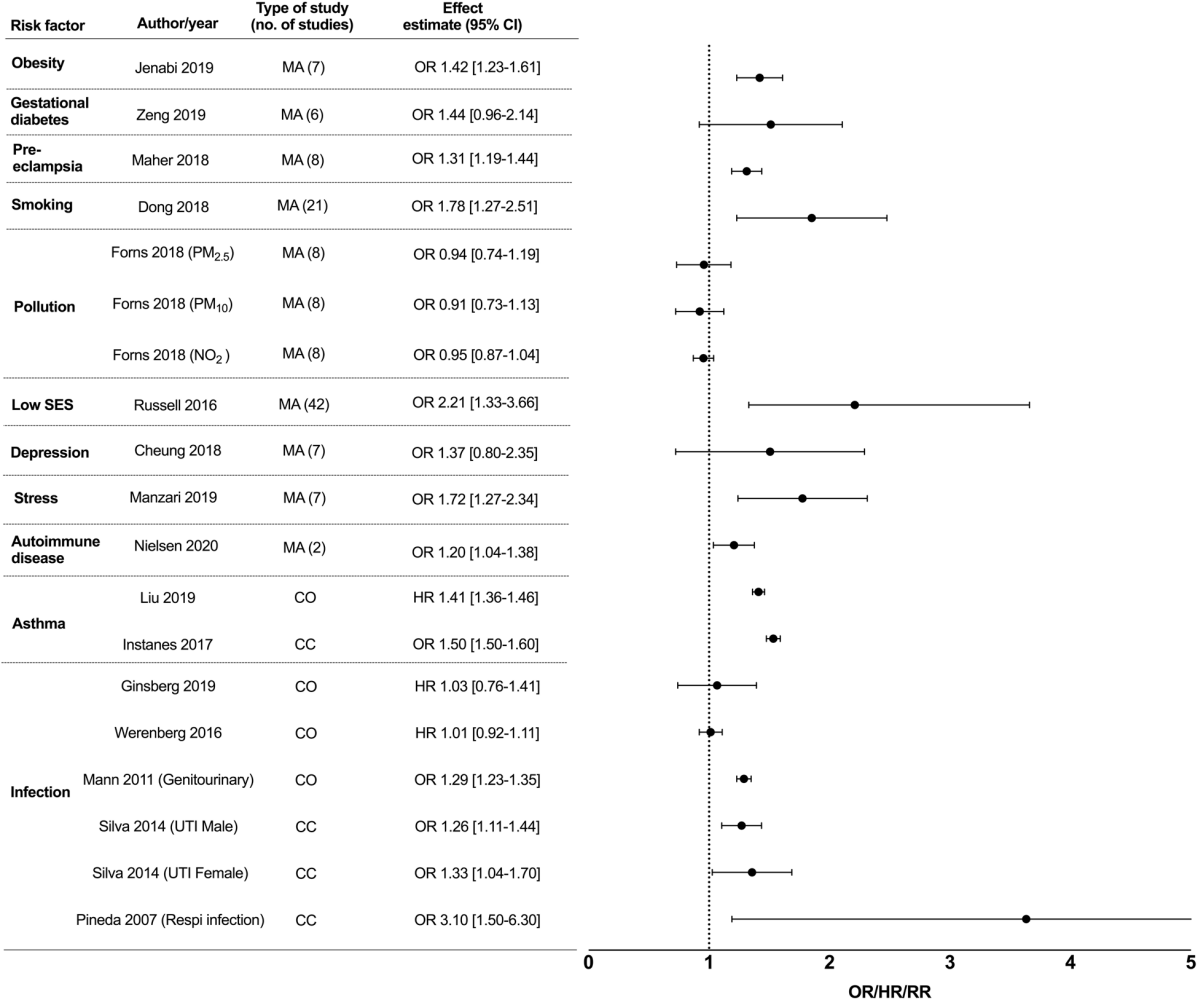
Epidemiological studies of maternal inflammatory states and attention deficit hyperactivity disorder (ADHD) in offspring. Maternal inflammatory states included in Y-axis (with columns including author names, year of publication, type of study, number of individual studies included in meta-analysis, effect estimate with 95% confidence interval (CI)). Effect estimate with 95% confidence interval reported in X-axis. In this forest plot, we only included the largest meta-analysis that examined the association of individual maternal inflammatory state and ADHD in offspring. For full data see Supplementary Fig. 2. SES = socioeconomic status, PM_2.5_ = particulate matter (particles with diameter of 2.5 micrometers or less), PM_10_ = particulate matter (particles with diameter of 10 micrometers or less), NO_2_ = nitrogen dioxide, UTI = urinary tract infection, Respi infection= respiratory infection, MA = meta-analysis, CC = case-control, CO = cohort, RR = relative risk, OR = odds ratio, HR = hazard ratio.

**Fig. 5:**
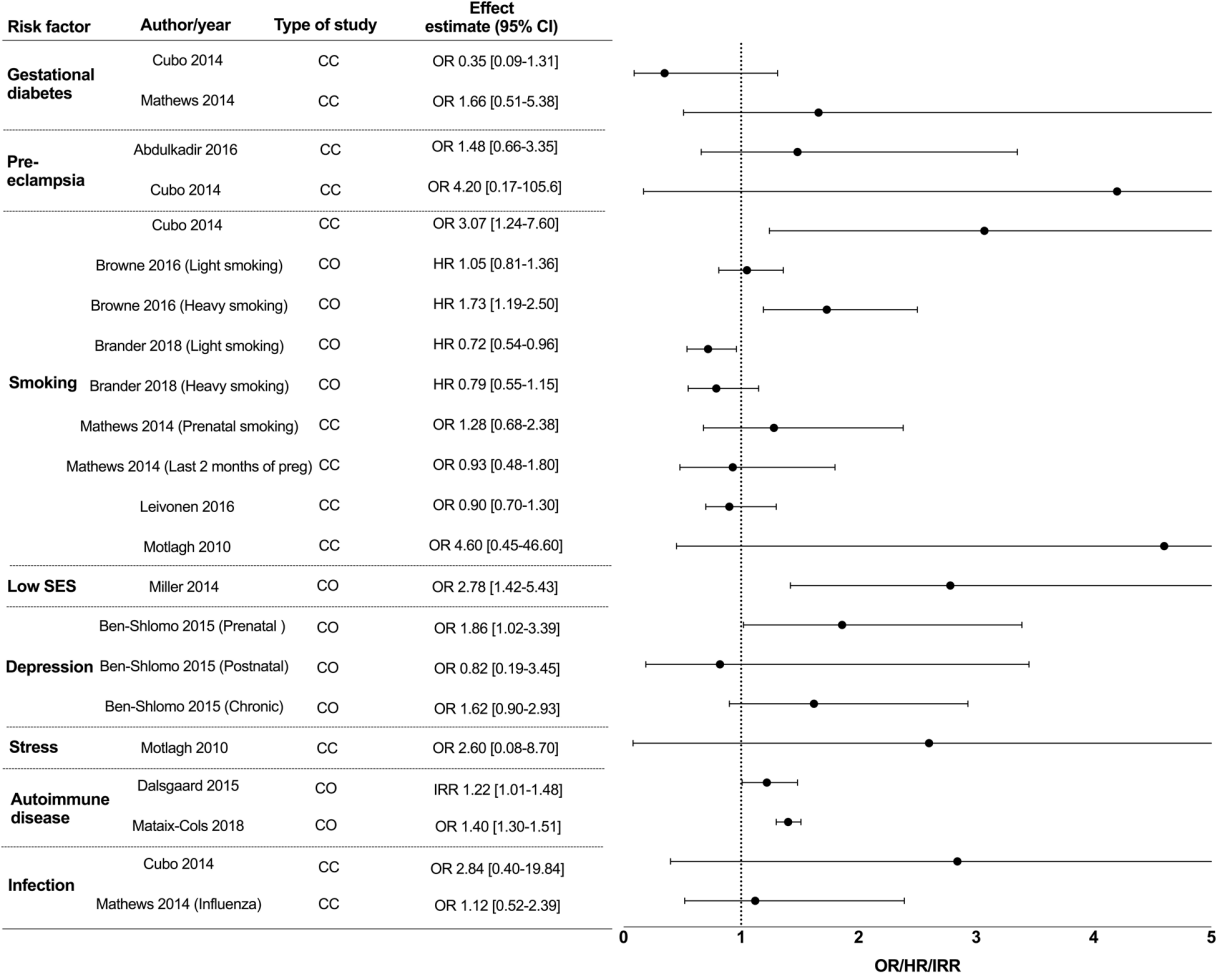
Epidemiological studies of maternal inflammatory states and Tourette syndrome in offspring. Maternal inflammatory states included in Y-axis (with columns including author names, year of publication, type of study, number of individual studies included in meta-analysis, effect estimate with 95% confidence interval (CI)). Effect estimate with 95% confidence interval reported in X-axis. SES socioeconomic status, Preg pregnancy, CC case-control, CO cohort, RR relative risk, OR odds ratio, HR hazard ratio, IRR incidence risk ratio.

A second search to look for individual studies examining these associations was performed. 4714 studies were identified from the databases and we included 12 cohorts, 13 case-control and 1 cross-sectional study (Fig. 2). Multiple studies from various countries examined association of socioeconomic status (SES) and ASD with different indicators used and a wide range of results found. We only included the 2 largest studies, which were from the United States of America and China as representative studies. 6 studies investigated an association between maternal asthma and ASD in offspring (5 case-control, 1 cohort). 2 studies examined an association between maternal asthma and ADHD in offspring (1 case-control, 1 cohort) and 5 studies examined an association between infection and ADHD in offspring (2 case-control, 3 cohort). Maternal inflammatory states and TS risk in offspring were analyzed in 6 cohort and 5 case-control studies. All these studies were included and summarized in forest plots (ASD- Supplementary Fig. 1, ADHD- Supplementary Fig. 2). If there were more than 1 meta-analysis found per maternal inflammatory state and neurodevelopmental disorder, we only included the largest meta-analysis in our final forest plots (ASD- Fig. 3, ADHD- Fig. 4, TS- Fig. 5) as a representative study to present the effect estimate of the association.

## Results

### Maternal inflammatory states and autism spectrum disorder in offspring

9 meta-analyses, 5 case-control, 2 cohort, and 1 cross-sectional study were included in the forest plot for ASD studies (Fig. 3, Supplementary Fig. 1). Maternal obesity, gestational diabetes, and pre-eclampsia were significantly associated with ASD in offspring (Supplementary Table 4)^9–19^. A linear dose-response relationship of maternal obesity with ASD was found, with a pooled RR of 1.16 (1.01–1.33) for each 5 kg/m^2^ increment in maternal body mass index (BMI)^9^. Maternal smoking was not associated with ASD^13,20–22^ although prenatal smoking OR 1.10 [1.03–1.17] had a marginal association with ASD^20^. Maternal exposure to pollution from particulate matter with a diameter of 2.5 micrometers or less (PM_2.5_) and nitrogen dioxide (NO_2_) was weakly associated with ASD, but studies investigating pollution from particulate matter with a diameter of 10 micrometers or less (PM_10_) generated mixed findings^23,24^. There was no evidence of an association between ozone (O_3_) and ASD^23,24^, nor was there a consistent association between trimester of exposure to pollutants and risk of ASD^23^.

Regarding socioeconomic status (SES), we included the 2 largest studies, which were from the United States of America (USA) (*n* = 557,689) and China (*n* = 616,940) as representative cohorts to demonstrate the association of SES and ASD in children^25,26^. Low SES in China was found to be associated with ASD (prevalence ratio low to high SES tertile of 2.27 [1.39–3.70]^26^), but in contrast, a reverse direction of effect between SES and offspring was reported in the USA (prevalence ratio low to medium SES tertile of 0.70 [0.64–0.76]^25^). Differences in ASD case ascertainment in studies are thought to explain these divergent results^25^. Studies that use data for children who receive services for ASD may be prone to “biased case ascertainment” and potentially under recognize ASD children in low SES families^27^. Thus, results may differ from countries with universal healthcare access and no economic barriers to obtaining diagnosis and services^27^. Maternal depressive disorders and stress were significantly associated with ASD, and subgroup analyses suggested an association between maternal stress and bereavement in the third trimester and increased risk of ASD^28,29^.

Maternal autoimmune disease during pregnancy, including thyroid disease and rheumatoid arthritis, was found to be an independent risk factor for ASD in offspring^30,31^. However, the 6 identified studies investigating the association between maternal asthma with ASD in offspring have produced mixed results^32–37^. Croen et al showed childhood ASD was associated with mothers’ asthma diagnosis in the first and second trimester but not in the third trimester or post delivery^34^. An association of maternal infection during pregnancy with ASD revealed the largest effect size for those requiring hospitalization for infection (OR 1.30 [1.14–1.50]^38^), site-specific bacterial infection during pregnancy (OR 1.18 [1.02–1.37]^38^) including genitourinary infections (OR 1.09 [1.02—1.16]^38^) and skin infections (OR 1.41 [1.13–1.77]^38^), and third-trimester infections (OR 1.20 [1.1–1.34]^38^). A smaller meta-analysis did not find urinary tract infection or respiratory infection to be associated with ASD in offspring^13^.

### Maternal inflammatory states and attention deficit hyperactivity disorder in offspring

A total of 9 meta-analyses, 3 case-control, and 4 cohort studies were included in the forest plot for ADHD studies (Fig. 4, Supplementary Fig. 2). Maternal obesity was associated with increased ADHD risk (BMI > 30 kg/m^2^, RR 1.64 [1.57–1.73]), compared to overweight state (BMI 25–30 kg/m^2^ RR 1.28 [1.17–1.40]^39^) suggesting a dose-dependent increase in risk^10^^,^^40^. Findings relating gestational diabetes and ADHD in offspring were mixed, but pre-eclampsia showed increased odds of ADHD^17,41,42^ (Supplementary Table 4). Maternal prenatal smoking was associated with ADHD in children^43–45^. Mothers who were heavy smokers (OR 1.75 [1.51–2.02]^43^) had increased odds of ADHD in offspring compared to mothers who were light smokers (OR 1.54 [1.40–1.70])^43–45^. Exposure to air pollution was not found to be significantly associated with childhood ADHD^46^.

Mothers with low SES had twice the odds (OR 2.21 [1.33–3.66]^47^) of having a child with ADHD, which authors propose may be mediated by maternal mental health disorders and maternal smoking during pregnancy. Maternal depression was not significantly associated with childhood ADHD^48^. Maternal stress was associated with ADHD in children; subjective self-report of stress but not objectively evaluated stress had increased association (OR 2.44 [1.23–4.85]^28^).

Maternal autoimmune diseases and asthma were significantly associated with ADHD^49–51^. Higher risk of ADHD was observed among offspring of mothers with asthma exacerbations and only exacerbations after delivery (HR 1.25 [1.08–1.44]^51^), suggesting that a more severe asthma phenotype results in greater risk to the offspring^51^. 3 studies showed an association between maternal genitourinary infections and childhood ADHD, and 1 study showed an association between maternal respiratory viral infection and ADHD^52–54^. However, maternal viral and bacterial infections requiring hospitalization during pregnancy were not associated with ADHD in the 2 cohort studies performed^53,55^.

### Maternal inflammatory states and Tourette syndrome in offspring

6 cohort and 5 case-control studies were included in the forest plots for TS studies (Fig. 5). No studies examined an association between maternal obesity and TS. Gestational diabetes and pre-eclampsia were not found to be associated with TS^56–58^. 6 studies investigated an association between maternal smoking and TS, but had conflicting results likely due to differences in methodology and sample characteristics^56,57,59–62^. Few studies showed significant association between maternal heavy smoking and TS/chronic tic disorder (CTD)^59,60^. No studies examined an association between pollution exposure in the mother and TS in the child. Low SES was associated with TS/CTD using a postnatal or combined factor composite score, however there was no association with prenatal composite factor scores^63^. The authors postulated that the postnatal SES score reflect prenatal adversities, which may become more apparent after the delivery of the child due to increased financial burden. Prenatal maternal depression, but not postnatal or chronic depression, nor maternal severe psychosocial stress, was associated with TS in offspring^62,64^.

Maternal autoimmune disease corresponded to an increased risk of TS, however individual risks involved with the most common autoimmune diseases did not reach statistical significance^65,66^. Maternal autoimmune disease was associated with a 29% increased incidence rate of TS in male offspring but no significant findings in female offspring, suggesting sex differences in susceptibility to maternal inflammation from autoimmune diseases^65^. No studies have examined an association between maternal asthma and TS in offspring. 2 cohort studies did not demonstrate an association between prenatal infection or maternal influenza at any time in pregnancy with TS in children^56,57^.

### Summary of study results

Heterogeneous maternal states are associated with an increased risk of common neurodevelopmental disorders in offspring. Although the maternal states are disparate and represent multi-factorial disease mechanisms, they are all associated with inflammation. Summarizing the combined data, maternal factors significantly associated with ASD include obesity, gestational diabetes, pre-eclampsia, pollution exposure to PM_2.5_ and NO_2_, depression, stress, autoimmune diseases, and infection; maternal factors significantly associated with ADHD include obesity, pre-eclampsia, smoking, low SES, stress, autoimmune diseases, asthma, genitourinary and respiratory viral infections; finally, maternal factors significantly associated with TS include low SES, depression and autoimmune diseases.

## Discussion

Neurodevelopmental disorders are genetically heterogeneous with increasing evidence that common neurodevelopmental disorders have shared genetic etiology from common genetic variants^67,68^. Developmental vulnerability genes have functions in neurogenesis and synaptogenesis with impaired neural circuitry established as the biological basis of neurodevelopmental disorders^69,70^. However, current modeling suggests that an individual’s genetic architecture and influence from the exposome, starting pre-conceptionally and continuing throughout life modify the expression of neurobehavioral outcomes^1,3,5,7^. In particular, maternal inflammation in pregnancy may program fetal inflammatory pathways and epigenetic machinery, potentially resulting in increased expression of neurodevelopmental disorders in childhood. The dose, type, intensity and timing of the immune response during pregnancy, likely influences fetal neurobehavior through impacts on specific developmental time course of different brain cells, regions and their underlying neural mechanisms^4,71^. Attempts to determine an association between timing of exposure to risk factors with childhood neurodevelopmental problems in epidemiology studies have been difficult^28,34,38,51,61^. Exposure to smoking in the first trimester appeared to increase the odds of TS and ADHD in the child, and exposure to maternal asthma in the first and second trimester is associated with childhood ASD. In contrast, the immune stress from maternal bacterial infections and negative life events seems greatest in the third trimester for both ASD and ADHD^28,34,38,61^. Evaluating the presence and timing of adverse or traumatic life events and assessments of related stress are not included in population databases and would need dedicated questionnaires or interviews, which are difficult to perform on a large scale. Additionally, studies that evaluate subjective depressive and stress symptoms are prone to recall and reporting bias thus skewing results. Dose-dependent effects of obesity and smoking have been demonstrated in studies^9,39,59^. However, population-based assessment of the severity of asthma, autoimmune disease, and depression in pregnancy is challenging, and may require surrogate markers including hospitalization stay or medication use. Prospective studies to examine longitudinal exposure to inflammatory states, with maternal-child genomic and immune profiling are needed to ascertain if the association is a result of cumulative immune stress or the direct impact of individual risk factors on susceptible periods of fetal brain development.

The cause of male predominance in ASD, ADHD, and TS is still not well delineated. Immunocompetent cells and inflammatory signaling, regulated by hormonal differences, direct brain masculinization during development^72^. Unfortunately, few studies examined how the same exposure results in gender-associated neurodevelopmental outcomes. For example, maternal autoimmune disease is associated with an increased incidence of male TS but not in females^65^. Animal MIA models also demonstrate male-dominant vulnerability to neurobehavioral problems in offspring, echoing the observations in human studies; however, the mechanisms that underlie the sexual dimorphism are unclear. Further study into the associations of maternal inflammatory states with neurodevelopmental disorders which include analysis by sex, and are sufficiently powered, are needed to uncover potential mechanisms.

Maternal immune activation may represent one of the convergent pathways in causing fetal neuroinflammation, resulting in increased expression of neurobehavioral phenotypes^3,7^. In ASD post-mortem brain, transcriptome analysis found synaptic and neuronal modules enriched in genome-wide association study (GWAS) genes were down-regulated, whereas up-regulated genes were inflammatory, not represented in genomic studies, and were thought to be secondary to environmental factors^73^. Further studies suggest contribution of epigenetics to this link between environmental factors and changes to immune pathways^74^. Epigenetic processes including DNA methylation, histone modifications, and chromatin remodeling are highly sensitive to environmental stimuli. In ASD brain, epigenetic modifications were found in genomic regions that encode microglial genes as well as candidate neurodevelopment genes^74^. A mouse model of maternal allergic asthma showed methylation alterations to microglia in the offspring corresponding to genes with altered expression in the ASD brain, providing evidence for the causative role for maternal immune activation and epigenetic dysregulation in fetal brain^8^. In human studies, DNA methylation modifications on immune genes were found in newborn cord blood from maternal exposure to gestational diabetes, obesity, and asthma, with follow-up studies showing persistent epigenetic alterations into adulthood^75,76^. Thus disruption of fetal epigenetic machinery by maternal immune activation could represent a key pathway for environmental contributions to the etiology of neurodevelopmental disorders with life-long effects^74^.

Maternal immune activation may act as a neurological disease primer through activated microglia^71^. Histological findings of altered microglia morphology and density and increased microglial gene transcription have been found in brain samples of adults with diverse neurodevelopmental disorders^73,77,78^. Microglia, resident innate immune cells in the brain, play a critical role in neurogenesis, myelination, synaptic pruning, and maintaining brain homeostasis^79^. Activated microglia acquire a pro-inflammatory phenotype, classically in response to infection but also cell injury and death in their surrounding. Microglia have a unique developmental origin, as they originate from the yolk sac and are rarely replaced by peripheral cells during normal development^79^. Thus, early-life perturbations to microglia have the potential to program microglia to an activated phenotype and undergo “priming” through epigenetic modifications, increasing later-life vulnerabilities to immune stimuli^71,79^. Microglial “priming” has been demonstrated in animal models where microglia have exaggerated inflammatory response to second “hits” from postnatal environmental stress^2,71,79^. Therefore, pre-conditioning of the microglia by maternal immune activation may increase susceptibility to neurobehavioral abnormalities in childhood.

While the role of genomic predisposition, epigenetic modification, and microglial activation in animal models have largely been established, the molecular mechanisms triggering and relaying maternal peripheral immune response to fetal microglial is less defined. Currently, cytokine signaling is proposed to mediate this peripheral-central cross-talk between mother and child^3,4^. Cytokines have pleiotropic roles, are critically involved in many important processes of brain development and in recent years, have been shown to directly mediate epigenetic modifications in DNA^74^. Release of pro-inflammatory cytokines through dysregulated innate or adaptive immune system, complement pathways, or through the involvement of maternal autoantibodies and altered blood-brain barrier integrity have been investigated with varying degrees of evidence^3^. In animal models, there is robust evidence for the role of toll-like receptors (TLR), pathogen recognition receptors, in maternal immune activation^7^. TLRs on maternal peripheral innate immune cells respond to various environmental factors including infectious and non-infectious stimuli resulting in transcriptional, histological, and behavioral deficits in offspring^3,7^. TLRs on peripheral innate immune cells are first responders to exogenous and endogenous ‘stress’ and ‘danger’ signals, triggering a cascade of downstream effects including cytokines, free radicals, and oxidative stress^7^. Peripherally produced proinflammatory cytokines communicate with microglia and perivascular macrophages to release neurokines through “leaky” subventricular organs or by stimulating afferent nerve pathways^80^. However, these mechanistic pathways are less established in humans and should be explored further to establish the first instigators of immune activation from diverse maternal states.

An inflammatory milieu during pregnancy also negatively affects other key pathways including neuroendocrine, sympathetic nervous system, metabolic and oxidative stress resulting in a spectrum of neurobehavioral deficits in children^3^ These individual pathways each have their unique role in the pathogenesis of neurobehavioral patterns in children; however, there is mounting evidence that immune signals modulate these pathways with multidirectional interactions between the systems^80^. For example, activated glial cells can directly inhibit serotonin production through the production of indoleamine 2,3,dioxygenase (IDO) which breaks down tryptophan to kynurenine, resulting in a reduced level of tryptophan (precursors of serotonin) and also indirectly by producing toxic by-products such as quinolinic acid which damage serotonergic neurons^80^. Innate immune activation can also trigger stress pathways through hypothalamus-pituitary-adrenal (HPA) axis via cytokine signaling and cyclooxygenase-2 (COX2) production^80^, and TLR4 can prime the HPA system towards hyper-reactivity to future stress-related insults^80^. Conversely, glucocorticoids may upregulate microglial TLR, amplifying future neuroinflammatory response^7^. Maternal factors such as depression and stress are postulated to increase gut permeability, resulting in increased translocation of maternal gut microbiome systemically to the placenta and fetal gut, which impacts fetal immunity via innate immune activation or inducing epigenetic changes through the release of short-chain fatty acids (bacterial metabolites)^81^. Therefore, the effects of ‘pro-inflammatory’ environmental factors are multifactorial and are modulated by epigenetic, neurogenic, metabolic, gut, and endocrine mechanisms.

There are limitations to the current human literature. Firstly, we chose meta-analyses with the largest number of cases analyzed to represent the association of individual maternal inflammatory states and neurodevelopmental disorder. This may have inadvertently missed out important studies not included in that specific meta-analysis. However, when we compared meta-analyses examining the same associations, there were no major discrepancies in the overall effect estimate (Supplementary Fig. 1, 2, Fig. 5). This shows that the results are robust despite different meta-analyses methodologies, lending strength to our overall result and hypothesis (Supplementary Table 4). Secondly, there is significant heterogeneity in the design of the studies included, with different patient selection, exposure, and outcome assessments, in addition to inherent differences in odds ratios, hazard ratios, and relative risks, thus study results and different measures of effect size reported should be interpreted with caution. A summary of individual study details is provided in Supplementary Table 4. Thirdly, although all of the factors associated in this review have demonstrated ‘inflammatory’ effects or associations, we recognize that some disorders are predominantly inflammatory (autoimmune, asthma, infection), others are mixed with neurogenetic, metabolic, and direct toxic effects (depression, obesity, diabetes, smoking, and pollution). Therefore, mechanisms of vulnerability are likely multifactorial, although we propose inflammation is one important unifying feature. Lastly, some studies may not have adjusted their results for relevant confounders such as prematurity or perinatal complications, which have increased prevalence in maternal inflammatory states (e.g. autoimmune diseases, infection, preeclampsia) and are also significant risk factors for neurodevelopmental disorders.

Our review supports the concept that heterogeneous maternal factors increase the expression of diverse neurodevelopmental disorders, and neurodevelopmental disorders should be genetically and epigenetically investigated beyond the boundaries of nosologically defined disorders. Broad-based evaluation of behavioral symptoms can help to unpick the complexities of gene-environment interaction and phenotypic expression. Future directions also should include moving away from traditional observational studies and the use of molecular genetically informed designs such as Mendelian randomization, to test causal hypotheses about prenatal exposure and offspring outcome.

Cumulative modeling of these risk factors is important to further understand the degree of impact, moderation, and synergism of multiple risk factors. Mothers of low SES status have increased rates of smoking, depression, stress, unhealthy diet, and sleep problems, which increase inflammatory status. A multiple exposure pregnant mouse model exposed to prenatal pollution and maternal stress in the last week of pregnancy resulted in behavioral deficits in male offspring^7^. On the other hand, isolated exposure to prenatal pollution induced maternal inflammation and microglial activation in the offspring of these mice but was not enough to elicit behavior changes^7^. This provides strong evidence that the combination of maternal pro-inflammatory risk factors in pregnancy increases developmental vulnerability in offspring^7^. In human studies, there is a lack of research investigating the association of two or more maternal risk factors with neurodevelopmental problems in offspring. Mann et al showed that children whose mothers had both genitourinary infection and pre-eclampsia were 53% more likely to have ADHD compared to those with neither exposure. In contrast, such analysis is lacking in other studies^52^. Lastly, more efforts to investigate the role of paternal and child inflammatory states, in conjunction with maternal factors, in the development of neurobehavioral deficits in the child are needed. Prenatal epigenetic modulation of genetic vulnerability in sperm and egg are likely relevant, in addition to the perinatal factors discussed here, as well as ongoing postnatal epigenetic modulation^74^.

The pathogenesis of neurodevelopmental disorders in children is still not fully understood. A complex interplay of genetic, epigenetic, and environmental factors throughout life is proposed. We demonstrate collective evidence that multiple maternal diseases, lifestyle, and environmental risk factors during pregnancy, which have in common inflammation, are associated with increased risk of ASD, ADHD, and to a lesser extent, TS. This supports the prevailing hypothesis that maternal inflammatory states contribute to immune activation during pregnancy, resulting in a range of neuropathologies including neuronal dysfunction, microglial activation, and a consequent spectrum of neurobehavioral phenotypes in children. Cumulative modeling of these maternal states would better reflect environmental exposure patterns and open up opportunities to explore commonalities, differences, and interactions of diverse maternal inflammatory states. An integrated approach to identify convergent pathways and molecular mechanisms of MIA in humans would provide new perspectives for better understanding, prevention, and early therapeutic intervention in high-risk pregnancies.

## Supplementary information

Supplementary Figure 1

Supplementary Figure 2

Supplementary table 1

Supplementary table 2

Supplementary table 3

Supplementary table 4
