# Supplementary material for: Maternal acute and chronic inflammation in pregnancy is associated with common neurodevelopmental disorders: a systematic review
Source: Transl Psychiatry. 2021 Jan 21;11:71. doi: 10.1038/s41398-021-01198-w (PMC7820474; doi:10.1038/s41398-021-01198-w)
Supplement: Supplementary file 3 — Supplementary table 1 [file 41398_2021_1198_MOESM3_ESM.docx]

Supplementary table 1

| *SEARCH STRATEGY* |
| --- |
| A literature search on PubMed and Embase databases was performed to identify studies published prior to 5^th^ June 2020, which examined associations of maternal inflammatory states with autism spectrum disorder (ASD), attention deficit hyperactivity disorder (ADHD) and Tourette Syndrome (TS). We used terms related to maternal, prenatal, inflammatory states (eg. obesity, gestational diabetes, pre-eclampsia, smoking, pollution, low socioeconomic status, depression, stress, autoimmune conditions, asthma and infection) and ASD, ADHD or TS (Supplementary table 2 for search terms). Definition of each inflammatory state used in this study is summarized in a table (Supplementary table 3 for definitions). Search for meta-analyses were performed first, and all meta-analyses, which met inclusion criteria, were included in the review. If there were no meta-analyses for an individual maternal inflammatory state and offspring neurodevelopment disorder, a search for individual studies was conducted. All studies published in English, irrespective of sample size, follow-up duration, or classification status were included. The search results from each database were combined, duplicates were removed, and reference lists of review articles and included studies were searched to identify additional eligible studies. |
| *STUDY ELIGIBILITY* |
| Studies were included if they met criteria as follows:   1. Meta-analyses, cohort or case control study design performed in mothers of offspring diagnosed with ASD (Asperger’s syndrome and pervasive developmental disorder-not otherwise specified included), ADHD or TS (chronic tic disorder included). Studies without a disease-free or exposure-free comparison group were excluded. 2. Maternal obesity, gestational diabetes, pre-eclampsia, smoking, pollution exposure, low socioeconomic status, depression, stress, autoimmune disease, asthma and infection established by one of the following: 1. Results from diagnostic tests, hospitalization or specialty clinic attendance 2. Diagnosis code documented in medical records 3. Self-report of diagnosis, exposure or personal details (eg. marital status, education level, postal code) through structured interview or questionnaires. Evaluation of medication use in mothers was excluded as it may reflect medication influence on fetal brain development rather than effect of the maternal inflammatory state. 3. ASD, ADHD, TS diagnosis in individual established by one of the following: 1. Diagnosis assigned by a medical professional or diagnosis code documented in records 2. Clinical assessments using structured screening or diagnostic tools, self-report questionnaires or structured interviews 3. Receipt of medication for the treatment of ADHD documented in prescription database. 4. Study includes reporting or provides data for calculation of effect size (eg. odds ratio, hazard ratio or relative risk). |
