# Supplementary material for: Maternal acute and chronic inflammation in pregnancy is associated with common neurodevelopmental disorders: a systematic review
Source: Transl Psychiatry. 2021 Jan 21;11:71. doi: 10.1038/s41398-021-01198-w (PMC7820474; doi:10.1038/s41398-021-01198-w)
Supplement: Supplementary file 4 — Supplementary table 2 [file 41398_2021_1198_MOESM4_ESM.docx]

Supplementary table 2: Search terms for review of studies of maternal inflammatory states and offspring autism spectrum disorder (ASD), attention deficit/hyperactivity disorder (ADHD) and Tourette Syndrome (TS)

| Databases | PubMed, Embase | | |
| --- | --- | --- | --- |
| Search Period | Earliest publication – 05/06/2020 | | |
| Search Terms | Maternal  AND | Inflammatory state  AND | neurodevelopmental disorder |
|  | (“mother” OR “pregnancy” OR “maternal” OR “prenatal” OR “perinatal”) | “obesity” OR “body mass index” | (“neurodevelopmental disorder” OR “autism spectrum disorder” OR “autism” OR “autistic” OR “ASD”) OR  (“attention deficit and disruptive behavior disorders’ OR “attention deficit hyperactivity disorder” OR “attention deficit disorder” OR “ADHD”) OR  (“tic disorder” OR “chronic tic disorder” OR “tourette syndrome” OR “tourette” OR “CTD” OR “TS”) |
|  |  | “diabetes mellitus” OR “gestational diabetes mellitus” OR “gestational diabetes” OR “GDM” |  |
|  |  | “hypertension, pregnancy induced” OR “pregnancy induced hypertension” OR “pre-eclampsia” OR “preeclampsia” |  |
|  |  | “smoking” OR “nicotine” |  |
|  |  | “air pollution” OR “pollution” OR “particulate matter” OR “nitrogen dioxide” OR “ozone” |  |
|  |  | “mood disorder” OR “depressive” OR “depression” OR “depressed” |  |
|  |  | “trauma and stressor related disorders” OR “stress” OR “trauma” OR “bereavement” OR “negative life events” OR “natural disaster” |  |
|  |  | “autoimmune diseases” OR “autoimmune” |  |
|  |  | “asthma” OR “hyperactive airway” |  |
|  |  | “infections” OR “infection” OR “bacteria” OR “virus |  |
|  | “socioeconomic factors” OR “socioeconomic status” OR “poverty” OR “income” OR “socioeconomic” OR “SES” | |  |
| Search #1 | Filter: human subjects, meta-analysis, systemic review  Using “explode” function for MESH/Emtree terms and including terms as keywords | | |
| Search #2 | If no meta-analysis or systemic review relevant for specific maternal risk factor and neurodevelopmental disorder, remove filter: human subjects  Using “explode” function for MESH/Emtree terms and including terms as keywords | | |
