# Supplementary material for: Maternal acute and chronic inflammation in pregnancy is associated with common neurodevelopmental disorders: a systematic review
Source: Transl Psychiatry. 2021 Jan 21;11:71. doi: 10.1038/s41398-021-01198-w (PMC7820474; doi:10.1038/s41398-021-01198-w)
Supplement: Supplementary file 5 — Supplementary table 3 [file 41398_2021_1198_MOESM5_ESM.docx]

Supplementary table 3: Definitions of inflammatory states

| Inflammatory state | Definition | Reference |
| --- | --- | --- |
| Obesity | Abnormal of excessive fat accumulation that may impair health. | ^1^ |
| Gestational diabetes | Any degree of glucose intolerance with onset or first recognition during pregnancy | ^2^ |
| Pre-eclampsia | New onset of hypertension at ≥ 20 weeks' gestation and proteinuria or new onset hypertension without proteinuria but with evidence of renal, hepatic or hematological dysfunction | ^3^ |
| Smoking | A smoker is someone who smokes any tobacco product, either daily or occasionally. |  |
| Pollution | Exposure to particulate matter with diameter of 2.5 micrometers or less (PM_2.5_) or diameter of 10 micrometers or less (PM_10_), nitrogen dioxide (NO_2_) and ozone (O_3_) |  |
| Depression | Depressive disorders or single/recurrent episodes, not including other mood/affective disorders like mania, bipolar or anxiety disorders |  |
| Stress | Psychological or environmental stress such as stressful life events, maternal bereavement, anxiety, depressive symptoms, traumatic events and natural disasters |  |
| Socioeconomic status | A measure of one’s combined economic and social status | ^4,5^ |
| Autoimmune disease | Conditions where:   1. The specific adaptive immune response is directed to the affected organ or tissue 2. Autoreactive T cells and/or autoantibodies are present in the affected organ or tissue and can transfer disease to healthy individuals/animals 3. Immunization with autoantigen induces the disease in animal models 4. Elimination of suppression of the autoimmune response prevents disease progression or even ameliorates clinical manifestation | ^6^ |
| Asthma | Asthma is a heterogenous disease, characterized by chronic airway inflammation. It is defined by history of respiratory symptoms such as wheeze, shortness of breath, chest tightness and cough that vary over time and in intensity, together with variable expiratory airflow limitation | ^7^ |
| Infection | Diseases caused by pathogenic miroorganisms, including bacteria, viruses, parasites or fungi |  |

1. Physical status: the use and interpretation of anthropometry. Report of a WHO Expert Committee. *World Health Organization technical report series.* 1995;854:1-452.

2. Gestational diabetes mellitus. *Diabetes care.* 2003;26 Suppl 1:S103-105.

3. ACOG Practice Bulletin No. 202: Gestational Hypertension and Preeclampsia. *Obstetrics and gynecology.* 2019;133(1):e1-e25.

4. Miller LL, Scharf JM, Mathews CA, Ben-Shlomo Y. Tourette syndrome and chronic tic disorder are associated with lower socio-economic status: findings from the Avon Longitudinal Study of Parents and Children cohort. *Developmental medicine and child neurology.* 2014;56(2):157-163.

5. Miller GE, Chen E, Fok AK, et al. Low early-life social class leaves a biological residue manifested by decreased glucocorticoid and increased proinflammatory signaling. *Proceedings of the National Academy of Sciences of the United States of America.* 2009;106(34):14716-14721.

6. Hayter SM, Cook MC. Updated assessment of the prevalence, spectrum and case definition of autoimmune disease. *Autoimmunity reviews.* 2012;11(10):754-765.

7. Hogan AD, Bernstein JA. GINA updated 2019: Landmark changes recommended for asthma management. *Annals of allergy, asthma & immunology : official publication of the American College of Allergy, Asthma, & Immunology.* 2020;124(4):311-313.
