# Supplementary material for: Maternal acute and chronic inflammation in pregnancy is associated with common neurodevelopmental disorders: a systematic review
Source: Transl Psychiatry. 2021 Jan 21;11:71. doi: 10.1038/s41398-021-01198-w (PMC7820474; doi:10.1038/s41398-021-01198-w)
Supplement: Supplementary file 6 — Supplementary table 4 [file 41398_2021_1198_MOESM6_ESM.docx]

Supplementary table 4: Studies of maternal inflammatory states and offspring autism spectrum disorder (ASD), attention deficit hyperactivity disorder (ADHD) and Tourette Syndrome (TS)

(a) Studies of maternal inflammatory states and autism spectrum disorder in offspring

| Maternal inflammatory state | Study ID | Individual studies | Study Design / Population | Location /  Study Period | Maternal exposure measure | ASD outcome in offspring | Covariates Controlled | Effect Estimate (95% CI) |
| --- | --- | --- | --- | --- | --- | --- | --- | --- |
| Obesity  15 individual studies | Wang  2016 | **Lyall 2011**  **Krakowiak 2012**  **Moss 2014**  **Suren 2014**  **Reynolds 2014**  **Gardner 2015**  **Xiang 2015** | Meta-analysis  7 studies  6 cohort  1 case-control  Total=509,107 | America (5),  Europe (2) | Self report (4), medical record (3), measure at first antenatal visit (1) | Parental report (2), M-CHAT (2), ADI-R, ADOS (2), ICD-9, ICD-10, DSM-IV (1) | Refer to meta-analysis  study table 1 | RR 1.36 [1.03-1.78] |
|  | Sanchez  2018 | **Lyall 2011**  Hendrix 2011  **Krakowiak 2012**  **Moss 2014**  **Suren 2014**  **Reynolds 2014**  Jo 2015  **Gardner 2015**  **Connolly 2016**  **Getz 2016**  **Li 2016** | Meta-analysis  11 studies  10 cohort  1 case-control  Total=497,585 | America (8),  Europe (3) | Self report (6), medical record (4), medical staff (2) | Parental report (4), ICD-9 (3), ICD-10, DSM-IV (1), ADI-R, ADOS (1), combined autism diagnostic interview, ADI-R, ADOS (1), M-CHAT (1), medical chart (1) | Refer to meta-analysis  study table 1 | OR 1.36 [1.08-1.70] |
|  | Lei  2019 | **Lyall 2011**  **Krakowiak 2012**  **Moss 2014**  **Reynolds 2014**  **Suren 2014**  **Gardner 2015**  **Xiang 2015**  **Connolly 2016**  **Getz 2016**  **Li 2016**  Casas 2017  Andersen 2017 | Meta-analysis  12 studies  9 cohort  3 case-control  Total=943,293 | America (7),  Europe (5) | Self report (6), medical record (6) | ICD-9 (3), ICD-10 (2), ADI-ADOS (2), M-CHAT (2), parental report (2), Read codes (1), childhood asperger syndrome test (1) | Refer to meta-analysis  study table 1 | OR 1.41 [1.19-1.67] |
|  | Li  2016 | Dodds 2011  **Krakowiak 2012**  **Moss 2014**  **Reynolds 2014**  **Suren 2014** | Meta-analysis  5 studies  4 cohort  1 case-control  Total=227,819 | America (3),  Europe (2) | Medical record (3), self report (2), research staff (1) | ICD-9, ICD-10 (2), ADI-R, ADOS (1), M-CHAT (1), parental report (1) | Refer to meta-analysis  study table 1 | OR 1.47 [1.24-1.74] |
| Gestational diabetes  12 individual studies | Wang  2017 | Hultman 2002  **Burstyn 2010**  **Dodds 2011**  Say 2016  Hadjkacem 2016 | Meta-analysis  5 studies  2 cohort  2 case-control  1 cross-sectional  Total= 351,452 | America (2), Europe (1), Africa (1), Asia (1) | Birth register/database (3), questionnaire (2) | ICD-9 (3), ICD-10 (1), CARS (1), DSM-IV (1), DSM-V (1) | Refer to individual studies | RR 1.49 [1.18-1.88] |
|  | Wan  2018 | **Lyall 2012**  **Piven 1993**  Connolly 2016 | Meta-analysis  3 studies  3 case-control  Total=105,874 | America (3) | Questionnaire (2), birth/delivery record (1) | ADI, ADOS (1), comprehensive multidisciplinary evaluation (1), questionnaire (1) | Refer to meta-analysis  study table 1  (Only included moderate to high quality studies) | RR 1.72 [1.34-2.21] |
|  | Xu  2014 | **Burstyn 2010**  **Dodds 2011**  **Lyall 2012** | Meta-analysis  8 studies  3 cohort  5 case-control  Total=536,325 | America (7),  Africa (1) | N/A (5), ICD-9 codes (3), ICD-10 code (1) | ADI-R (3), ADOS (3), ICD-9 (3), DSM-IV (2), CARS (2), ICD-10 (1), | Refer to meta-analysis  study table 1 | Cohort studies  RR 1.43 [1.13-1.79] |
|  |  | **Piven 1993**  Juul-dam 2001  Croen 2005  Brimacombe 2007  Elhameed 2011 |  |  |  |  |  | Case-control studies  OR 2.23 [1.38-3.60] |
| Pre-eclampsia  19 individual studies | Dachew  2018 | **Glasson 2004**  **Larsson 2005**  **Buschmayer 2009**  **Bursytn 2010**  **Mann 2010**  **Dodds 2011**  **Langridge 2013**  **Polo-Kantola 2014**  **Walker 2015**  **Xiang 2015** | Meta-analysis  10 studies  4 cohort  6 case-control  Total=1,166,307 | America (5),  Europe (3),  Australia (2), | Medical records (6), ICD-9 code (1), self report (1), research data (1), perinatal database (1), birth registry (1) | ICD-9 (6), ICD-10 (4), DSM-IV (2), DSM-III R (1), ICD-8 (1), ADI-R (1) | Refer to meta-analysis  study table 1 | OR 1.32 [1.20-1.45] |
|  | Jenabi  2019 | **Glasson 2004**  **Larsson 2005**  **Buschmayer 2009**  **Burstyn 2010**  **Mann 2010**  **Dodds 2011**  **Moore 2012**  **Langridge 2013 Polo-Kantola 2014**  Raz 2015  **Walker 2015**  **Xiang 2015**  Getahun 2017 | Meta-analysis  13 studies  6 cohort  7 case-control  Total= 7,561,696 | America (6), Europe (5), Australia (2) | NR | ICD-9 (6), ICD-10 (4), DSM-IV (2), DSM-V (2), DSM (1), ADOS, ADI-R (1) | Refer to meta-analysis  study table 1 | Cohort studies  RR 1.30 [1.20-1.41] |
|  |  |  |  |  |  |  |  | Case-control studies  OR 1.36 [1.12-1.60] |
|  | Maher  2018 | Deykin 1980  **Mason-Brothers 1990**  **Matsuishi 1999**  **Glasson 2003**  **Larsson 2005**  **Buchmayer 2009**  **Burstyn 2010**  **Mann 2010**  Lyall 2012  Mrozek-Budzyn 2013  **Walker 2015** | Meta-analysis  11 studies  3 cohort  8 case-control  Total=399,571 | America (6),  Europe (3), Asia (1), Australia (1) | Medical report/self report (3), ICD-9 (3), medical/delivery record (3), birth registry (1), ICD-10 (1) | ICD-9 (3), ICD-10 (3), ICD-8 (1), DSM III (1), DSM III R (1), ADI-R/ADOS (1), maternal report (1), ≥ 1 symptom of impaired relatedness to the environment, stereopathy and impaired language development (1) | Refer to study supplementary online content | OR 1.37 [1.07-1.75] |
|  | Xu  2018 | Gilberg 1983  **Mason-Brothers 1990**  **Matsuishi 1999**  **Glasson 2004**  **Larsson 2005**  Stein 2006  **Buchmayer 2009**  **Burstyn 2010**  **Mann 2010**  **Moore 2012**  **Walker 2015** | Meta-analysis  12 studies  6 cohort  5 case-control  Total= 6,302,167 | America (5), Europe (3), Asia (2), Australia (1) | NR | ICD=9 (5), DSM-III (3), ICD-8 (2), ICD10 (2), ADI-R, ADOS (1) | Refer to meta-analysis  study table 1 | OR 1.43 [1.31-1.55] |
|  | Wang  2017 | **Glasson 2004**  **Mann 2010**  **Burstyn 2010** | Meta-analysis  3 studies  2 cohort  1 case-control  Total= 308,826 | America (2), Australia (1) | Database/records (2), ICD-9 (1) | ICD-9 (2), DSM (1) | Refer to individual studies | RR 1.50 [1.04-2.18] |
| Smoking  30 individual studies | Rosen  2015 | Juul-Dam2001  **Hultman 2002**  **Williams 2003**  **Larrson 2005**  **Maimburg 2006**  **Larsson 2009**  **Burstyn 2010**  **Hvidtjorn 2011**  **Dodds 2011**  **Haglund 2011**  Volk 2011  **Kalkbrenner 2012**  **Lee 2012**  **Tran 2013**  Visser 2013 | Meta-analysis  15 studies  3 cohort  11 case-control  1 case-cohort  Total= 1,705,134 | America (6),  Europe (9) | Timing of maternal smoking: prenatal (9), at birth (2), after birth (5) | NR | Refer to meta-analysis  study table 1 | OR 1.02 [0.93-1.12] |
|  | Tang  2015 | **Hultman 2002**  **Larsson 2005**  **Maimburg 2006**  **Bilder 2009**  **Larsson 2009**  **Burstyn 2010**  **Dodds 2010**  **Haglund 2011**  **Kalkbrenner 2012**  **Lee 2012**  **Tran 2013**  Nilsen 2013  **Mrozek-Budzyn 2013**  **Xiang 2015** | Meta-analysis  15 studies  6 cohort  9 case-control  Total=1,810,258 | America (5),  Europe (10) | Collected by midwives at registration for antenatal care/visits (4), questionnaire (2), recorded by check boxes at the beginning and the end of pregnancy (2), reported at first antenatal visit (1), parent-report collected for pregnancy when child 1-3 years (1), collected on admission to hospital for delivery (1), birth certificate data (1), medical birth registry (1), from birth records and certificate records (1), N/A (1) | Medical registry (6), medical record (4), administrative database (1), surveillance-ascertained (1), clinician review of medical and school record (1), paediatric developmental specialist evaluation (1), parent-report collected by follow-up questionnaire (1) | Refer to meta-analysis  study table 1 | OR 1.02 [0.93-1.13] |
|  | Wang  2017 | **Hultman 2002**  **Bilder 2009**  **Burstyn 2010**  Mann 2010  Zhang 2010  **Dodds 2011**  **Haglund 2011**  Hadjkacem 2016  Say 2016 | Meta-analysis  9 studies  3 cohort  5 case-control  1 cross-sectional  Total= 521,865 | America (4), Europe (2), Asia (2), Africa (1) | Database/register (5), questionnaire (3), ICD-9 (1) | ICD-9 (4), ICD-10 (3), DSM-IV (2), DSM IV-TR (1), DSM-V (1), DSM III (1), CARS (1), Gilberg & Gilberg criteria (1) | Refer to individual studies | RR 1.02 [0.88-1.19] |
|  | Jung  2017 | **Williams 2003**  **Larsson 2005**  **Maimburg 2006**  **Bilder 2009**  **Larsson 2009**  **Burstyn 2010**  **Dodds 2011**  **Haglund 2011**  **Hvidtjorn 2011**  Volk 2011  **Kalkbrenner 2012**  **Lee 2012**  **Mrozek-Budzyn 2013**  Roberts 2013  **Tran 2013**  Visser 2013  Schimdt 2014  Gao 2015  **Kalkbrenner 2015**  Schieve 2015  Talbott 2015  **Xiang 2015** | Meta-analysis  22 studies  7 cohort  15 case-control  Total= 2.624.888 | America (12),  Europe (10) | Timing of maternal smoking: prenatal (7), at birth (4), after birth (9) | Medical record (16), direct evaluation (4), parental report (2) | Refer to meta-analysis supplementary table | OR 1.16 [0.97-1.40] |
| Pollution  25 individual studies | Chun  2020 | Kalkbrenner 2010  Volk 2011  **Becerra 2013**  **Jung 2013**  Roberts 2013  **Volk 2013**  Windham 2013  **Gong 2014**  **Raz 2015**  **Kalkbrenner 2015**  Talbott 2015  Guxens 2016  Gong 2017  Kim 2017  Al-Hamdan 2018  Chen 2018  Goodrich 2018  Kerin 2018  Kalkbrenner 2018  Raz 2018  Ritz 2018  Yousefian 2018  Kaufman 2019  Pagalan 2019 | Meta-analysis  25 studies  3 cohort  21 case-control 1 ecological | America (17), Europe (7), Asia (4) | Pollution exposure before, during and after pregnancy  PM_2.5_ (25), PM_10_ (23),  NO_2_ (16), ozone (9) | Refer to study table A1 | Refer to meta-analysis appendix | PM_2.5_  OR 1.06 [1.01-1.11] |
|  |  |  |  |  |  |  |  | PM_10_  OR 1.01 [0.99-1.03] |
|  |  |  |  |  |  |  |  | NO_2_  OR 1.02 [1.01-1.04] |
|  |  |  |  |  |  |  |  | O_3_  OR 1.00 [1.00-1.01] |
|  | Lam  2016 | **Becerra 2013**  **Jung 2013**  **Volk 2013**  **Gong 2014**  **Raz 2015**  **Kalkbrenner 2015** | Meta-analysis  6 studies  1 cohort  5 case-control  Total= 150,988 | America (4), Europe (1), Asia (1) | Residential address, PM_2.5_ (3), PM_10_ (6) | Database (2), ADI-R (2), ADOS (1), SDQ (1), ATAC, CATSS (1), record based surveillance (1) | Refer to individual studies | Per 10-ug/m^3^ increase in PM_2.5_ exposure  OR 2.32 [2.15-2.51] |
|  |  |  |  |  |  |  |  | Per 10-ug/m^3^ increase in PM_10_ exposure  1.07 [1.06-1.08] |
| Low socio-economic status  2 individual studies | He  2018 | | Cohort  Total= 616,940 | China  April-May 2006 | Socioeconomic status  Family income (low, middle, high), adults’ education (low, middle, high), housing tenure (owner vs rent vs others)- divided into 3 SES tertiles | ICD10 F84 | Age, ethnicity, education, housing tenure | Low: high income tertile 2.27 [1.39-3.70]  (inverted for comparison) |
|  | Durkin  2010 | | Cross-sectional  Total= 557,689  ASD= 3680 | USA  2002-2004 | Socioeconomic status  Income above federal poverty level, bachelor’s degrees, median household income – weighted and divided into 3 SES tertiles | DSM-IV=TR | Nil | Low: medium tertile prevalence ratio  0.70 [0.64-0.76] |
| Depression  3 individual studies | Ayano  2019 | Daniels 2008  Havid 2013  Rai 2013 | Meta-analysis  3 studies  1 cohort  2 case-control  Total=672,431 | Europe (3) | ICD (3) | ICD-10 (3), ICD-9 (2) | Refer to meta-analysis  study table 1 | OR 1.62 [1.32-1.99] |
| Stress  9 individual studies | Manzari  2019 | Li 2009  Zhang 2010  Rai 2012  Hamade 2013  Class 2014  George 2014  Gao 2015  Roberts 2016 | Meta-analysis  9 studies  5 cohort  4 case-control  Total=2,346,200 | Europe (4), Asia (4), America (1) | Unhappy emotional state- questionnaire (2), maternal bereavement- nationwide registry (2), severe life events (death, serious accident/illness)- nationwide registry (1), exposure to rare and common life event – questionnaire (1), excessive mental stress- questionnaire (1), depressive symptoms (1), sexual, emotional, physical abuse (1) | DSM-IV (1), CARS score ≥30 (1), DSM-IV + CARS score ≥30 (1), ICD-10 (1), ADI-R (1), inpatient/outpatient diagnosis (1), children in psychiatric hospital or in receipt of outpatient care because of  ASD (1), structured neuropsychiatric assessment (1), diagnosis identified through national Health Records and the Pupil Level Annual Schools  Census (PLASC) (1) | Refer to meta-analysis supplementary table | OR 1.64 [1.15-2.34] |
| Autoimmune disease  15 individual studies | Chen  2016 | Comi 1999  Sweeten 2003  Croen 2005  **Mouridsen 2007**  **Keil 2010**  Andersen 2014  Lyall 2012  Lyall 2014  Brown 2015  Khaiman 2015 | Meta-analysis  10 studies  1 cohort  9 case-control  Total=961,986 | America (4),  Europe (4), Asia (1) | Questionnaire (4), ICD-8/ICD-10 (2), ICD-9 (1), ICD (1), TPO-Ab/TSH/fT4 (1), N/A (1) | ICD-10 (3), DSM-IV (2), ICD-9 (2), ICD-8 (1), ADI/ADOS (1), questionnaire (1), recruited from East Tennessee Chapter of the Autism Society of America (1) | Refer to meta-analysis  study table 1 | OR 1.34 [1.23-1.46] |
|  | Zhu  2020 | **Mouridsen 2007**  Atladottir 2009  **Keil 2010**  Vinet 2015  Tsai 2017  Rom 2018  Croen 2019 | Meta-analysis  7 studies  4 cohort  3 case-control  Total= 4,568,326 | Europe (4), America (2), Asia (1) | Medical records in registry (5), ICD-10 (3), ICD-8 (2), health insurance database (1), self-report (1) | ICD-10 (4), ICD-9 (3), ICD-8 (2), ADOS, ADI-R (1) | Refer to meta-analysis  study table 1 | Rheumatoid arthritis  OR 1.39 [1.16-1.67] |
|  |  |  |  |  |  |  |  | Systemic lupus erythematosus  OR 1.32 [0.64-2.72] |
| Asthma  6 individual studies | Gong  2019 | | Case-control  ASD=22,894, control=228,940 | Sweden  Born 1992-2007 | National patient register (diagnostic codes), medical birth register (tick box for asthma/lung disease), Swedish prescribed drug register (SPDR) (≥2 dispensed packages of inhaled corticosteroids, leukotriene antagonist, fixed-dose B2-ICS combinations, or ≥3 packages of ICS, LTRA, fixed-dose B2-ICS combinations, inhaled B2-agonist within 12months) | ICD-9 code 299, ICD-10 code all F84s | Control: age, country, sex-matched  Parity, maternal factors: smoking during pregnancy, civil status at year of child birth, country of birth, parental age at child birth, highest education between parents, maternal BMI at first antenatal visit | OR 1.43 [1.38-1.49] |
|  | Hisle-Gorman 2018 | | Case-control  ASD=8760 control=26,280 | USA  Oct 2000-Sept 2013 | ICD-9 and ambulatory pharmaceutical records | ICD-9 code at 2 separate encounters between Oct 2000 and Sept 2013 (2-18 years of age) | Control: age, sex, child’s enrolment timeframe-matched | OR 1.49 [1.29-1.74] |
|  | Croen  2005 | | Case-control  ASD=320 control=2100 | USA  Born January 1995-June 1999 | ICD-9-CM | ICD-9-CM code 299.0, 299.8 | Control: sex, birth year, hospital of birth- matched  Maternal factors: age, education, race/ethnicity, plurality | OR 1.60 [1.20-2.20] |
|  | Lyall  2014 | | Case-control  ASD=560 control=391 | USA  Started 2003 | Questionnaire | ADOS, ADI-R | Control: gender, regional area, age-matched  Maternal factors: breastfeeding, medication use for asthma and allergies, smoking, race, education, insurance status at delivery | OR 1.00 [0.69-1.44] |
|  | Croen  2019 | | Case-control  ASD=663 control=915 | USA (multi-sites)  Born  2003-2006 | Telephone interview, questionnaire | ADOS, ADI-R | Child gender, current household income, maternal age, race, education | OR 1.26 [0.99-1.60] |
|  | Langridge  2013 | | Cohort  Total=383,153  ASD=452 | Australia  Born 1984-1999 | Midwives’ notification system | DSM-IIIR, DSM-IV, DSM-IV-TR | Birth year, sociodemographic factors | OR 1.41 [0.98-2.04] |
| Infection  18 individual studies | Jiang  2016 | **Glasson 2004**  Maimburg 2006  Buchmayer 2009  Atladottir 2010  **Dodds 2011**  Abdallah 2012  Atladottir 2012  Langridge 2013  Mamidala 2013  Visser 2013  Zerbo 2013  Zerbo 2013  Duan 2014  Fang 2015  Lee 2015 | Meta-analysis  15 studies  2 cohort  13 case-control  Total=4,625,342 | Europe (9), America (3), Australia (2), Asia (1) | ICD-10 (6), ICD-9 (4), ICD-8 (4), questionnaire (4), ICD-9-CM (1), medical record (1), interview (1), N/A (1) | ICD-10 (7), ICD-8 (3), ICD-9 (2), ICD-9-CM (2), DSM (2), DSM-IV(2), ADOS, ADI-R (2), DSM-IIIR (1), CBCL (1) | Refer to meta-analysis  study table 1 | OR 1.13 [1.03-1.23] |
|  | Wang  2017 | **Glasson 2004**  Mann 2010  Hadjkacem 2016 | Meta-analysis  3 studies (UTI)  1 cohort  1 case-control  1 cross sectional  Total= 90,037 | America (1), Australia (1), Africa (1) | Research database (1), questionnaire/medical record (1), ICD-9 (1) | DSM (1), DSM V (1), ICD-9 (1) | Refer to individual studies | RR 0.95 [0.70-1.30] |
|  |  | Zhang 2010  **Dodds 2011**  Hadjkacem 2016 | 3 studies (Respiratory infection)  1 cohort  1 case-control  1 cross sectional  Total=130,024 | America (1), Asia (1), Africa (1) | Questionnaire (2), perinatal database (1), medical record (1) | DSM V (1), ICD-9/10 (1), ICD-10, CARS (1) | Refer to individual studies | RR 2.64 [0.78-8.86] |

(b) Studies of maternal inflammatory states and attention deficit hyperactivity disorder in offspring

| Maternal inflammatory state | Study ID | Individual studies | | Study Design / Population | Location /  Study Period | Maternal exposure measure | ADHD outcome in offspring | Covariates Controlled | Effect Estimate (95% CI) |
| --- | --- | --- | --- | --- | --- | --- | --- | --- | --- |
| Obesity  16 individual studies | Jenabi  2019 | Burg 2007  Mikkelsen 2017  **Rodriguez 2008**  **Rodriguez 2010**  Buss 2012  **Chen 2014**  **Jo 2015**  **Li 2016**  Andersen 2018  Kong 2018 | | Meta-analysis  10 studies  10 cohorts  Total=1,464,097 | Europe (7), USA (3) | Measured (5), self report (5) | DSM-V criteria (4), ICD-10 code (3), SDQ (2), ICD-9 code (1), RB2 (1), doctor/other health professional (1) | Socioeconomic status, smoking during pregnancy, maternal psychiatric diagnoses, year of birth, mother’s country of birth, parental age, gestational age, birth weight, child sex, maternal education, and maternal age at delivery | OR 1.42 [1.23-1.61] |
|  |  |  |  |  |  |  |  |  | HR 1.65 [1.55-1.76] |
|  | Sanchez  2018 | **Rodriquez 2010**  Buss 2012  **Jo 2015**  Van Mil 2015  **Li 2016**  Messer 2016  Pugh 2015, 2016 | | Meta-analysis  7 studies  6 cohort,  1 case-control  Total=15,466 | America (5), Europe (2) | Self report (3), medical records (2), research staff (2) | Attention. CBCL parent (3), ICD-9 code (2), attention, TRF (1), DSM-IV criteria (1), previous diagnosis, parent report (1) | Refer to meta-analysis  study table 1 | OR 1.62 [1.23-2.14] |
|  | Li  2020 | **Rodriguez 2008**  **Rodriguez 2010**  Hinkle 2013  **Chen 2014**  Casas M 2017 | | Meta-analysis  5 studies  5 cohort  Total=696,919 | America (4), Europe (1) | Self report (3), medical record (1), birth register (1) | DSM-IV criteria (3), ICD-9, ICD-10, ADHD medication (1), SDQ, RB2 (1), previous diagnosis (1) | Refer to meta-analysis  study table 1 | RR 1.64 [1.57-1.73] |
| Gestational diabetes  9 individual studies | Zeng  2019 | **Nomura 2012**  Schmitt 2012  Mimouni-Bloch 2013  Li 2016  Say 2016  Xiang 2018 | | Meta-analysis  6 studies  3 cohort  2 case-control  1 cross sectional  Total= 349,955 | America (3), Asia (2), Europe (1), | Questionnaire (2), ICD-9 code (1), interview (1), medical record (1), mother report diagnosed by doctor (1) | DSM-IV criteria (2), ICD-9 code (2), ICD-10 code (1), diagnosed by medical centre (1) | Refer to study | OR 1.44 [0.96-2.14] |
|  | Zhao  2019 | **Nomura 2012**  Li 2014  Bytoft 2017  Daraki 2017 | | Meta-analysis  4 studies  4 cohort  Total= 2516 | Europe (2), America (1), Asia (1) | Blood glucose test (1), screen at 24-28weeks (1), interview (1), Danish diabetic association (1), | Standardised child scale (2), child psychiatric interview (1), self report use of medication (1) | Refer to meta-analysis  study table 1 | RR 2.00 (1.42-2.81) |
| Pre-eclampsia  8 individual studies | Maher  2018 | Gustafsson & Kallen 2011  Mann & McDermott 2011  Amiri 2012  Halmoy 2012  Ketzer 2012  Getahun 2013  Golmirzaei 2013  Silva 2014 | | Meta-analysis  8 studies  4 cohort  4 case-control  Total= 1,414,709 | Asia (3), Europe (2), America (2), Australia (1) | Medical birth registry (2), ICD-9-CM code (1), ICD-9 code (1), self-report (2), questionnaire (1), NICU record (1), midwifes’ notification system (1) | DSM-IV criteria (3), DSM-IV-TR criteria (1), DSM-III criteria (1), DSM-III-R criteria (1), ICD-9 code (1), ICD-10 code (1), ICD-9-CM ≥ separate visits or a diagnosis on 1 visit and ≥2 refills of ADHD specific medication (1), stimulant medication according to ICD-9 and DSM-IV criteria | Refer to meta-analysis  supplementary online content | OR 1.31 [1.19-1.44] |
| Smoking  34 individual studies | He  2017 | **Rodriguez 2005**  **Nigg 2007**  Biederman 2009  **Ball 2010**  Anselmi 2010  **Sciberras 2011**  **Langley 2012**  **Sagiv 2013**  **Jaspers 2013**  Skoglund 2014  **Melchior 2015**  **Obel 2016** | | Meta-analysis  12 studies  12 cohorts  Total=17,304 | Europe (6), America (4), South America (1), Australia (1) | Maternal smoking during pregnancy | DSM-IV criteria (5), DSM-III-R criteria (2), ICD-10 code (2), SDQ (2), medical record (1), parent report (1), ADHD meds (1) | Refer to meta-analysis  study table 1 | RR 1.58 [1.33-1.88] |
|  | Huang  2018 | **Milberger 1996**  **Mick 2002**  **Kotimaa 2003**  **Knopik 2005**  **Schmitz 2006**  Wakschlag 2006  **Nigg 2007**  **Yoshimasu 2009**  **Ball 2010**  **Lindblad 2010**  Hutchinson 2010  Obel 2011  **Sciberras 2011**  **Langley 2012**  **Sagiv 2013**  **Jaspers 2013**  **Silva 2014**  **Melchior 2015**  **Obel 2016**  Gustavson 2017 | | Meta-analysis  20 studies  15 cohort  5 case-control  Total=2,998,059 | Europe (11), America (7), Australia (2), South America (1), Asia (1) | Maternal smoking during pregnancy: interview (9), questionnaire (3), medical birth register (2), recorded at recruitment (2), preventative child healthcare files (1), midwives notification record (1), self report (1), collected by midwife at first antenatal visit (1), | DSM-IV (9), DSM-III-R (4), ICD-10 code (4), SDQ (2), RB2 (1), N/A (1) | Refer to meta-analysis  study table 1 | OR 1.60 [1.45-1.76] |
|  | Dong  2018 | **Milberger 1996**  Milberger 1998  **Mick 2002**  **Kotimaa 2003**  **Knopik 2005**  **Rodriguez 2005**  Braun 2006  **Schmitz 2006**  Froehlich 2009  Obel 2009  **Yoshimasu 2009**  Brion 2010  Gustafsson 2010  **Lindblad 2010**  Nomura 2010  **Langley 2012**  Schmitt 2012  **Silva 2014**  Han 2015  **Melchior 2015**  Joelsson 2016 | | Meta-analysis  27 studies  Total= 3,076,173 | America and Europe (24), Asia (2), Australia (1) | Refer to individual studies | Refer to individual studies | Parental psychiatric history, socioeconomic status | OR 1.78 [1.27-2.51] |
| Pollution  8 individual cohorts | Forns  2018 | | | 8 cohorts  Total=29,127 | Europe (8)  1992-2008 | Land-use regression models using home address at birth and road traffic load and intensity | ADHD traits within clinical range:  A-TAC, CBCL11/2-5, SDQ, ADHD-DSM-IV criteria  Refer to study table | Refer to study table | PM_2.5_  OR 0.94 [0.74-1.19] (per 5ug/m^3^ increase) |
|  |  |  |  |  |  |  |  |  | PM_10_  OR 0.91 [0.73-1.13] (per 10ug/m^3^ increase) |
|  |  |  |  |  |  |  |  |  | NO_2_  OR 0.95 [0.87-1.04]  (per 10ug/m^3^ increase) |
| Low socio-economic status  42 individual studies | Russell  2016 | | Scahill 1999  Andres 1999  Graetz 2001  Biedermann 2002  Omoy 2003  Kotimaa 2003  Franz 2003  Ford 2004  St Sauver 2004  Barry 2005  Counts 2005  Cornejo 2005  Montiel-Naava 2005  Khamis 2006  Schneider 2006  Visser 2007  Bauermeister 2007  de Ridder 2007  Dopfner 2008  Lee 2008  Al Hamed 2008  P’Olak 2009  Li 2009  Wagner 2009  Pastura 2009  Roberts 2009  Yoshimasu 2009  Bener 2009  Anselmi 2010  Flouri 2010  Siddique 2011  Sciberras 2011  Duric 2011  Apouey 2011  Boe 2012  Lingenini 2012  Russell 2013  Sagiv 2013  de la Barra 2013  Kvist 2013  Pires 2013  Getahun 2013 | Meta-analysis  42 studies  7 cohort  9 case-control  25 cross-sectional 1 ecological  Total= 1,358,080 | Europe (15), USA (12), South America (7), Asia (6), Australia (2) | Socioeconomic status measures: income, education, occupation, single parent, index  Refer to meta-analysis  study table 2 | Refer to meta-analysis  study table 2 | Refer to individual studies | OR 2.21 [1.33-3.66] |
| Depression  7 individual studies | Cheung  2018 | | Morrison & Stewart 1971  Lahey 1988  Nigg 1996  Chronis 2003  Goldstein 2006  Kepley & Ostrander 2007  Schatz 2012 | Meta-analysis  7 studies  7 case-control  Total=597 | America (7) | BDI, semi-structured clinical interviews | CBCL, Conner’s’ parenting rating scale-revised, BASC-2 | Refer to meta-analysis study | OR 1.37 [0.80-2.35] |
| Stress  7 individual studies | Manzari  2019 | | Lee 2006  Kim 2009  Li 2010  Martini 2010  Motlagh 2010  Class 2014  Park 2014 | Meta-analysis  7 studies  4 cohort  3 case-control  Total= 1,758,906 | Asia (3), Europe (3), America (1) | Maternal bereavement – nationwide register (2), self perceive distress- questionnaire (1), maternal stress/depression (1), marital adaptation/ satisfaction, life event perception- questionnaire (1), psychosocial stressors about home environment, parental relationship, emotional supports, employment, financial status, physical health, and  legal issues- interview (1) | Diagnosis by expert psychiatrist (1), DSM-IV criteria (1), ICD-10 code and ADHD med (1), inpatient and outpatient diagnosis (1), composite international diagnostic interview (1), DISC-IV criteria (1), SADS-PLV (1) | Refer to meta-analysis  study supplementary table | OR 1.72 [1.27-2.34] |
| Autoimmune disease  2 individual studies | Nielsen  2020 | | Nielsen 2017  Nielsen 2020 | Meta-analysis  2 studies  2 cohort  Total=1,046,690 | Australia (1), Europe (1) | Any autoimmune disease- state hospital records (1), national inpatient and outpatient hospital register (1) | ICD-10AM in hospital records or ≥ 1 stimulant auth or RX (1), ICD-10 code in psychiatric or national register (1) | Refer to meta-analysis  study table 3 | OR 1.20 [1.04-1.38] |
| Asthma  2 individual studies | Liu  2019 | | | Cohort  Total=961,202  Exposure=83,266 | Denmark  Born 1997-2012, followed up to 20 years of age | ICD-8 code 493, ICD-10 code J45, J46 or redemption of ≥2 anti-asthma medication prescription (B2-agonist, inhaled glucocorticoids, leukotriene receptor antagonist, anti-IgE treatment) | ICD-8 code 308.01, ICD-10 code F90, F98.8 or redemption of ADHD medication (amfetamine, dexamphetamine, methylphenidate, atomoxetine, dexmethylphenidate, lisdexamfetamine) | Maternal factors: age at delivery, parity, smoking during pregnancy, mental disorders before pregnancy, cohabiting status, place of residence at delivery, low social class at delivery, large family size, gender of child, calendar year of birth | HR 1.41 [1.36-1.46] |
|  | Instanes  2017 | | | Nested case-control study  ADHD=47,944 control=2,274,713 | Norway  Born 1967-2012 | Medical birth registry of Norway | Receive ADHD medication 2004-2012 (atomoxetine, amphetamine) >3years old at last prescription | Year of birth, parity, maternal age at birth, maternal education, maternal marital status, birth weight, gestational age | OR 1.50 [1.50-1.60] |
| Infection  5 individual studies | Ginsberg 2019 | | | Cohort  Total=1,066,956  Exposure=11,895 | Sweden  Born 1992-2009 | Maternal infection during pregnancy requiring hospitalisation:  Respiratory, nervous system, intestinal, skin, joint, genital, hepatic, urinary tract infection ICD-9 and ICD-19 discharge codes | ICD-9 code 314,  ICD-10 code F90 | Offspring sex, birth order, parental age, parental highest education, country of birth, lifetime history of severe mental illness, substance use problem, attempted suicide and criminal convictions, stratified by maternal identity, sibling comparisons** | HR 1.03 [0.76-1.41]** |
|  | Werenberg  2016 | | | Cohort  Total=89,146, exposure=29,861 | Denmark  Born 1992-2002 | Genitourinary, persistent viral infections, prolonged cough, diarrhea), interview | ICD-10 DF90.0-DF90.0 or redeemed prescription for stimulant medication | Maternal factors: education, age, psychiatric disease, smoking, stress, pre pregnancy BMI, number of children in household, calendar year of birth | HR 1.01 [0.92-1.11] |
|  | Mann  2011 | | | Cohort  Total=84,721 ADHD=7911 | USA  Born 1996-2002 | Infection of genitourinary tract during pregnanc:  Trichomoniasis, gonorrhoea, chlamydia, vulvovaginal candidiasis, urinary tract infection, vaginitis, cervicitis, chorioamnionitis/pelvic inflammatory disease/upper reproductive tract infections, ICD-9 codes | ICD-9 codes 314.00, 314.01 | Nil | OR 1.29 [1.23-1.35] |
|  | Silva  2014 | | | Case-control  ADHD=12,991 control=30,071 | Australia  Born since 1980 (<25 years of age) | Urinary tract infection during pregnancy: midwives notification system | Prescribed stimulant medication using DSM-IV-TR or ICD-10 criteria (August 2003-  Dec 2007) | Control: year of birth, gender, socioeconomic status- matched  Maternal factors: age, marital status, SEIFA, first pregnancy, threatened abortion, pre-eclampsia, induced labour, fetal distress, cord prolapse, child’s gestation, birth weight, Apgar at 5minsyear of birth | Male  OR 1.26 [1.11-1.44] |
|  |  |  |  |  |  |  |  |  | Female  OR 1.33 [1.04-1.70] |
|  | Pineda  2007 | | | Case- control ADHD=200  control =286 | Columbia  2000 | Respiratory tract infection during pregnancy: questionnaire | DSM-IV criteria | Gender, child’s age, school grades | OR 3.10 [1.50-6.30] |

(c) Studies of maternal inflammatory states and Tourette syndrome in offspring

| Maternal inflammatory state | Study ID | Study Design / Population | Location /  Study Period | Maternal Exposure | TS/CTD outcome in offspring | Covariates Controlled | Effect Estimate (95% CI) |
| --- | --- | --- | --- | --- | --- | --- | --- |
| Gestational diabetes | Cubo  2014 | Nested case-control  total=407 TD=64 control=89 | Spain  March 2007-Dec 2009 | Questionnaire | TD  DSM-IV-TR criteria | Control: age, gender-matched  Family history of tics, body mass index, comorbid neuropsychiatric disturbance | OR 0.35 [0.09-1.31] |
|  | Mathews  2014 | Nested case-control  TS=50 control=5968 | United Kingdom  Born April 1991-  Dec 1992 | Questionnaire | TS  DSM-IV-TR criteria | Nil | OR 1.66 [0.51-5.38] |
| Pre-eclampsia | Abdulkadir  2017 | Case-control  CTD=586 control=527 | 24 sites in USA, Europe, South Korea  Sept 2010- June 2014 | Questionnaire | TS/CTD  DSM-IV-TR criteria | Age, gender | OR 1.48 [0.66-3.35] |
|  | Cubo  2014 | Nested case-control  Total=407 TD=64 control=89 | Spain  March 2007-Dec 2009 | Questionnaire | TD  DSM-IV-TR criteria | Control: age, gender-matched  Family history of tics, body mass index, comorbid neuropsychiatric disturbance | OR 4.2 [0.17-105.6] |
| Smoking | Cubo  2014 | Nested case-control  Total=407 TD=64 control=89 | Spain  March 2007-Dec 2009 | Prenatal smoking: questionnaire | TD  DSM-IV-TR criteria | Control: age, gender-matched  Family history of tics, body mass index, comorbid neuropsychiatric disturbance | OR 3.07 [1.24-7.6] |
|  | Browne  2016 | Cohort  Total=73,073  TS/CT=427 | Denmark  Born 1996-2002 | Prenatal smoking: questionnaire | TS/CT  ICD-10 code F95.1, F95.2 | Birth year, sex, maternal age, parity, maternal psychiatric disorders, socioeconomic status, consumption of alcohol, coffee, smoking hashish, binge drinking, partner smoking, gestational age and birth weight | Light smoking  HR 1.05 [0.81-1.36]  ] |
|  |  |  |  |  |  |  | Heavy smoking  HR 1.73 [1.19-2.50 |
|  | Brander  2018 | Cohort  Total=3,026,861  TS/CTD=5597 | Sweden  Born 1973-2003, followed up until 2013 | Prenatal smoking: multi-generation registrar | TS/CTD  ICD-8 code 206.2, ICD-9 code 307C, ICD-10 code F95.0, F95.1, F95.2, F95.8, F95.9 | Sex, year of birth, age of parents, parity, full sibling comparison** | Light smoking  HR 0.72 [0.54-0.96]** |
|  |  |  |  |  |  |  | Heavy smoking  HR 0.79 [0.55-1.15]** |
|  | Mathews  2014 | Nested case-control  TS=50 control=5968 | United Kingdom  Born April 1991-  Dec 1992 | Smoking before pregnancy and last 2 months of pregnancy:  questionnaire | TS  DSM-IV-TR criteria | Socioeconomic status | Smoking before pregnancy  OR 1.28 [0.68-2.38] |
|  |  |  |  |  |  | Maternal age, socioeconomic status, parity, less/more than adequate weight gain, medications of vomiting in 2^nd^ trimester, alcohol use, cannabis use | Smoking during last 2 months of pregnancy  OR 0.93 [0.48-1.80] |
|  | Leivonen  2016 | Case-control  TS=767 control=2698 | Finland  Diagnosed between 1991-2010 | Prenatal smoking:  Finnish medical birth register | ICD-10 code F95.2, ICD-9 code 3072D | Control: date of birth, sex, place of birth-matched  Parental psychiatric history, parental age, birth weight, gestational age, maternal socioeconomic status | OR 0.90 [0.70-1.30] |
|  | Motlagh  2010 | Case-control  Total=222 TS=45 control=65 | USA,  1993-2003 | Prenatal smoking: interview | TS  DSM-IV criteria | Gender | OR 4.60 [0.45-46.60] |
| Low socioeconomic status | Miller  2014 | Cohort  Total=7152 TS=50 control=5968 | United Kingdom  Born 1991-1992, followed up until 13 years of age | Individual level: educational level, occupation, housing, car ownership, crowding index, household-based measure, self reported financial difficulties and index of multiple deprivation | TS/CT  DSM-IV criteria | Nil | Combined factor score (prenatal and post natal)  OR 2.78 [1.42-5.43 |
|  |  |  |  |  |  |  | Prenatal factor score  OR 1.40 [0.80-2.48] |
|  |  |  |  |  |  |  | Postnatal factor score  OR 2.09 [1.38-3.47] |
| Depression | Ben-Shlomo  2015 | Cohort  Total=14,541  TS/CT=122 | United Kingdom  Born April 1991-Dec 1992 | Self reported depression measures at 18/32 weeks gestation, 8 weeks and 8 months postnatal (prenatal, postnatal, chronic) | TS/CT  DSM-IV-TR criteria | Maternal or paternal age, socioeconomic status factor, parity | Prenatal depression  OR 1.86 [1.02-3.39] |
|  |  |  |  |  |  |  | Postnatal depression  OR 0.82 [0.19-3.45] |
|  |  |  |  |  |  |  | Chronic depression  OR 1.62 [0.90-2.93] |
| Stress | Motlagh  2010 | Case-control  Total=222 TS=45 control=65 | USA,  1993-2003 | Interview, ≥4 ordinal level of stress severity scale | TS  DSM-IV criteria | Gender | OR 2.60 [0.08-8.70] |
| Autoimmune disease | Dalsgaard  2015 | Cohort  Total=1,116,255  TS=2442 | Denmark  Born 1990-2006, followed up until 2010 | ICD-8, ICD-10 code (31 conditions | TS  ICD-10 code F95.2 | Gender, calendar time, age, place of birth, parental diagnosis of TS, parental psychiatric diagnoses other than TS, parental age at birth | IRR 1.22 [1.01-1.48] |
|  | Mataix-Cols  2018 | Cohort  TS/CTD=7083  control= 7,409,510 | Sweden  Born 1940-2007, followed up to 2013 | ICD codes (40 conditions) | TS/CTD  ICD-8, ICD-9, ICD-10 codes | Nil | OR 1.40 [1.30-1.51] |
| Infection | Cubo  2014 | Nested case-control  Total=407 TD=64 control=89 | Spain  March 2007-Dec 2009 | Questionnaire | TD  DSM-IV-TR criteria | Control: age, gender-matched  Family history of tics, body mass index, comorbid neuropsychiatric disturbance | OR 2.84 [0.40-19.84] |
|  | Mathews  2014 | Nested case-control  TS=50 control=5968 | United Kingdom  Born April 1991-  December 1992 | Influenza: questionnaire | TS  DSM-IV-TR criteria | Nil | OR 1.12 [0.52-2.39] |

ASD= autism spectrum disorder, ADHD= attention deficit hyperactivity disorder, TS= Tourette Syndrome, TD= tic disorder, CTD= chronic tic disorder, DSM= diagnostic and statistical manual of mental disorders, ICD= international classification of diseases, M-CHAT= modified checklist for autism in toddlers, ADI-R= autism diagnostic interview-revised, ADOS= autism diagnostic observation schedule, CARS= childhood autism rating scale, SDQ= strengths, difficulties questionnaire, A-TAC= autism-tics, attention deficit/hyperactivity disorder (AD/HD) and other comorbidities inventory, CBCL= child behaviour checklist, RB2= rutter teacher questionnaire, BASC-2= behaviour assessment system for children, SADS/PLV= schedule for affective disorders and schizophrenia for school-age children-present and lifetime version (SADS-PLV), SES= socioeconomic status, PM_2.5_= particulate matter that have diameter less than 2.5 micrometers, PM_10_= particulate matter that have diameter of less than 10 micrometers, NO_2_= nitrogen dioxide, O_3_= ozone, CATSS= child and adolescent twin study in Sweden, TPO-Ab= thyroid peroxidase antibody, TSH=thyroid-stimulating hormone, fT4= free thyroxine, ICS= inhaled corticosteroids, B2= beta2-adrenergic agonist, LTRA= leukotriene receptor anatagonist, , BDI=Beck depression inventory
